# Focus On: Neurotransmitter Systems

**Published:** 2011

**Authors:** C. Fernando Valenzuela, Michael P. Puglia, Stefano Zucca

**Keywords:** Maternal alcohol exposure, prenatal alcohol exposure, fetal alcohol syndrome disorders, pregnancy, developmental disorders, central nervous system, neurotransmitter systems, amino acids, biogenic amines, animal models

## Abstract

Neurotransmitter systems have been long recognized as important targets of the developmental actions of alcohol (i.e., ethanol). Short- and long-term effects of ethanol on amino acid (e.g., γ-aminobutyric acid and glutamate) and biogenic amine (e.g., serotonin and dopamine) neurotransmitters have been demonstrated in animal models of fetal alcohol spectrum disorders (FASD). Researchers have detected ethanol effects after exposure during developmental periods equivalent to the first, second, and third trimesters of human pregnancy. Results support the recommendation that pregnant women should abstain from drinking—even small quantities—as effects of ethanol on neurotransmitter systems have been detected at low levels of exposure. Recent studies have elucidated new mechanisms and/or consequences of the actions of ethanol on amino acid and biogenic amine neurotransmitter systems. Alterations in these neurotransmitter systems could, in part, be responsible for many of the conditions associated with FASD, including (1) learning, memory, and attention deficits; (2) motor coordination impairments; (3) abnormal responsiveness to stress; and (4) increased susceptibility to neuropsychiatric disorders, such as substance abuse and depression, and also neurological disorders, such as epilepsy and sudden infant death syndrome. However, future research is needed to conclusively establish a causal relationship between these conditions and developmental dysfunctions in neurotransmitter systems.

This article reviews recent research on the short- and long-term effects of developmental ethanol^1^ (i.e., alcohol) exposure on brain chemical (i.e., neurotransmitter) systems. The article focuses on studies that were performed with tissue from animal models, including rats, mice, guinea pigs, and primates. It is noteworthy that prenatal development in rats and mice corresponds to the first and second trimesters of human pregnancy, whereas the first week of neonatal life corresponds to the third trimester. In guinea pigs and primates, intrauterine development more closely corresponds to the first, second, and third trimesters of human pregnancy. It also is important to keep in mind that the studies in this research area are quite heterogeneous in several respects, including the timing, duration, and route of ethanol exposure; the levels of ethanol that were achieved in blood; and the techniques used to assess the effects of ethanol exposure. Regarding blood ethanol levels, it should be emphasized that the legal intoxication limit for driving is 0.08 g/dl and that, in some cases, developmental exposures to much higher ethanol levels were required to produce significant effects (see table 1). Ethanol concentrations near 0.4 g/dl are typically lethal in individuals who do not regularly drink significant amounts of ethanol and have not developed tolerance to its depressant effects on brain activity. Therefore, care must be exercised when interpreting the results of studies that have used high concentrations of ethanol. This article first provides background information on neurotransmitter systems and their roles in normal central nervous system development and neurodevelopmental disorders. It then reviews studies on the actions of ethanol on two types of neurotransmitter systems: amino acids and biogenic amines. For the most part, the article reviews research published in the past decade. The reader is referred to more comprehensive review articles for additional information (Berman and Hannigan 2000; Costa et al. 2000; Goodlett et al. 2005; Valenzuela et al. 2008; Weinberg et al. 2008). Figure 1 illustrates some of the mechanisms by which developmental ethanol exposure could impair chemical neurotransmitter systems.

## Neurotransmitter Systems and Normal Central Nervous System Development

**Figure 1:**
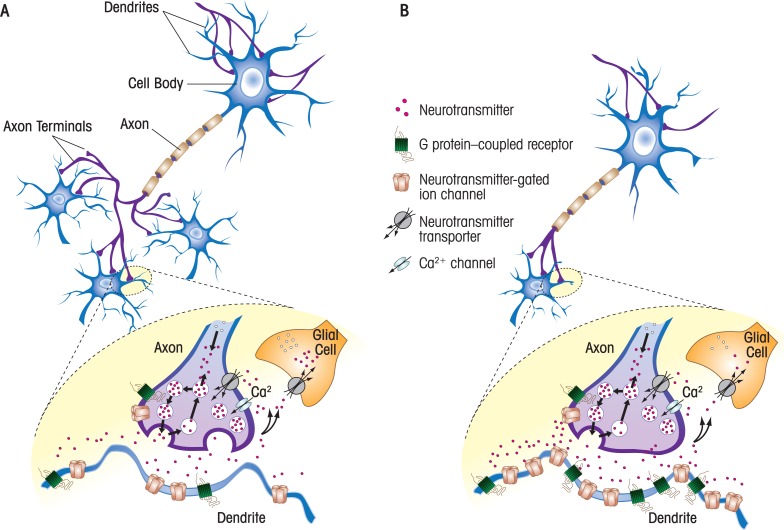
Potential sites of action of developmental ethanol exposure on neurotransmitter systems. **A)** Shown in the upper panel is a schematic representation of the components of a neuron, including the cell body and the ends of projections (axons and axon terminals) that release neurotransmitter onto branch-like projections (dendrites) in adjacent neurons. Note that the axons make synaptic connections with dendrites from three adjacent neurons. Shown in the lower panel is a synapse in more detail. In axonal terminals, neurotransmitters are synthesized and packaged into synaptic vesicles. Release of the neurotransmitter is triggered by influx of Ca^2+^ via voltage-gated Ca^2+^ channels. Neurotransmitter release can be modulated by neurotransmitter-gated ion channels and G protein–coupled receptors expressed in axonal terminals. Neurotransmitters act by activating neurotransmitter-gated ion channels and G protein–coupled receptors expressed in target neurons (either at postsynaptic or extrasynaptic locations). One mechanism by which the action of the neurotransmitter can be terminated is by reuptake into the axonal terminal or neighboring glial cells via neurotransmitter transporters. **B)** Upper panel: developmental ethanol exposure could affect a given neurotransmitter system by decreasing the number of neurons, dendrite and axonal length and/or number, and/or modifying the number and/or efficacy of synapses. Lower panel: developmental ethanol exposure can affect any of the components involved in neurotransmission. In this hypothetical example, ethanol exposure decreased neurotransmitter release, and this led to a compensatory increased in the levels of postsynaptic and extrasynaptic neurotransmitter receptors in the target neuron.

Efficient communication among large numbers of brain cells (i.e., neurons) is necessary for the normal functioning of the nervous system. A central mechanism of neuronal communication involves the release of neurotransmitters that bind to specialized receptors on the target cell, changing its activity. Although neuropeptides are an important category of neurotransmitters involved in neuronal communication and the developmental actions of ethanol, they are beyond the scope of this article. This article will focus on chemical neurotransmitters, which can be divided into three classes: (1) amino acids (e.g., γ-aminobutyric acid [GABA], glycine, and glutamate); (2) biogenic amines (e.g., serotonin, dopamine, norepinephrine, epinephrine, and histamine); and (3) other (e.g., acetylcholine, adenosine triphosphate, and adenosine). This review focuses on the actions of ethanol on the GABA, glutamate, serotonin, and dopamine neurotransmitter systems in the developing brain. Neurons synthesize these neurotransmitters and package them in vesicles that typically are localized at the ends of projections known as axons. Neuronal activation causes the release of neurotransmitters from the axonal terminals onto branch-like projections in adjacent neurons, which are called dendrites. Dendrites contain small thorn-like protrusions known as dendritic spines. Axonal terminals and dendritic spines meet at specialized points of contact, called synapses, which mediate a significant portion of information exchange between neurons (see figure 1). Neurotransmitter receptors are not only expressed in target cell dendrites but also in axonal terminals, where these receptors regulate neurotransmitter release (see figure 1). Reuptake into the axonal terminal or neighboring support cells (i.e., glial cells) or enzymatic breakdown decreases synaptic levels of the chemical neurotransmitter, terminating its action.

Neurotransmission in the mature central nervous system relies on the proper assembly of synapses during development, a process that requires multiple steps. Development of the nervous system begins with the recruitment of progenitor cells (precursors of neurons and glial cells) into a specialized structure called the neural plate. The neural plate then folds to form the neural tube, which subdivides in a complex manner. The anterior and posterior regions of the neural tube ultimately give rise to the brain and spinal cord, respectively. Within the neural tube, progenitor cells are transformed into neuronal and glial cells. Immature neurons migrate to their final locations and gradually acquire axons, dendrites, and synapses. Information exchange between axons and dendrites determines whether synapses are maintained or eliminated, and chemical neurotransmitters play important roles in each of these neuronal developmental steps. Even before synapses are formed, GABA and glutamate regulate progenitor cell proliferation, migration, and differentiation (Manent and Represa 2007). These neurotransmitters could be released from growing axons via reverse action of neurotransmitter transporters that normally pump neurotransmitters into the axonal terminal. In immature neurons, glutamate and GABA contribute to the maturation of dendrites and axons and also are directly involved in the generation and refinement of synaptic contacts (Cline and Haas 2008). Neurotransmitter systems have different properties during development and maturity. For instance, neurons throughout the developing central nervous system generate oscillatory electrical activity that plays a central role in the construction of neuronal networks. These unique properties contribute to the formation and maturation of neuronal circuits and also make immature neuronal networks particularly susceptible to genetic or environmental insults (Ben-Ari 2008).

## Role of Alterations in Neurotransmitter Systems in Neurodevelopmental Disorders

Multiple studies have identified neurotransmitter systems as major substrates of neurodevelopmental disorders, including autism, Down syndrome, and fetal alcohol spectrum disorders (FASD). Although these conditions have different causes, they are all characterized by altered neuronal communication that can be explained by underlying deficiencies in synapse development. Ethanol exposure during development has been shown to cause long-lasting defects in both the structure and function of synapses. Several mechanisms could underlie these persistent defects. Death, abnormal migration, or arrested maturation in a population of neurons will deprive their targets from receiving appropriate synaptic inputs, allowing for abnormal synaptic connections to be formed.

Even if synapses are formed properly, ethanol can affect the normal progression of their developmental program, which involves stabilization of functional synapses and pruning of unneeded synapses. Importantly, these synaptic refinement processes are regulated by chemical neurotransmission and require equilibrium (also known as homeostasis) between inhibitory and excitatory influences (Ramocki and Zoghbi 2008). This equilibrium can be altered by genetic defects, such as in the case of Fragile X syndrome, which is characterized by deficits in excitatory synaptic transmission mediated by the amino acid transmitter glutamate; or exposure to toxic agents such as ethanol, which, as discussed below, produces complex effects on the balance between excitatory and inhibitory neurotransmitters. In response to these perturbations, developing neurons attempt to restore equilibrium with compensatory changes that often involve increases or decreases in the function of proteins (e.g., neurotransmitter receptors) involved in chemical neurotransmission. These compensatory changes are not always able to restore equilibrium, and this slows down or accelerates developmental programs, causing abnormal assembly of neuronal circuits and long-lasting alterations in chemical neurotransmission. The precise chain of events leading from developmental insult-induced alterations in neurotransmitter systems to persistent neurochemical alterations during adulthood is currently unknown. In the case of developmental ethanol exposure, this issue is very difficult to study because drinking during pregnancy can occur in many different patterns—for instance, single versus repeated exposure, ingestion of low versus high amounts of ethanol, and exposure during early versus late pregnancy. The studies reviewed below are quite heterogeneous, involving diverse animal models, patterns of developmental ethanol exposure, and study end points. Significant progress has been made in this area of research in recent years, as reviewed below.

## GABA

**Figure 2:**
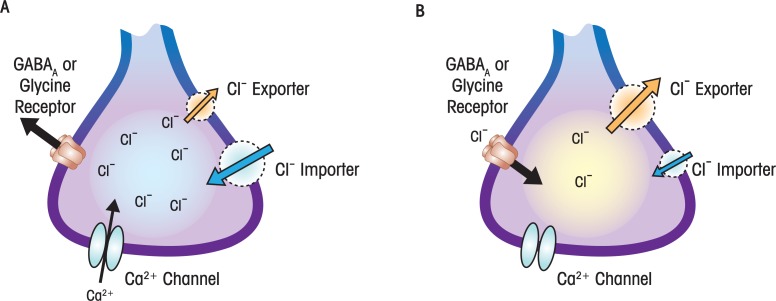
γ-Aminobutyric acid (GABA)_A_ receptors stimulate immature neurons and inhibit mature neurons. **A)** In immature neurons, intracellular Cl^−^ concentrations are higher than in mature neurons. This is a consequence of low expression of a Cl^−^ exporter (potassium/chloride cotransporter type 2; KCC2) and high expression of a Cl^−^ importer (sodium/potassium/chloride cotransporter type 1; NKCC1). Activation of GABA_A_ receptors causes Cl^−^ flux out of the cell, which makes the membrane potential more positive, leading to activation of Ca^2+^ channels. **B)** In mature neurons, intracellular Cl^−^ concentrations are low. This is a consequence of high expression of a Cl^−^ exporter and low expression of a Cl^−^ importer. Activation of GABA_A_ receptors causes Cl^−^ flux into the cell, which makes the membrane potential more negative. Ca^2+^ channels are not activated under these conditions. The unique properties of GABA_A_ receptors during development make them especially vulnerable to ethanol (see text).

GABA is synthesized from glutamate by the enzyme glutamate decarboxylase and is the main inhibitory transmitter in the mature mammalian brain. Two classes of receptors—the GABA_A_ and GABA_B_ receptors—mediate the actions of this neurotransmitter. Most studies related to FASD have focused on GABA_A_ receptors, which are GABA-activated ion channels that are permeable to chloride ions (Cl^−^). When these receptors are activated by GABA in mature neurons, Cl^−^ flows into the cell making the membrane potential more negative and thereby decreasing excitability of the neuron (see figure 2). However, when these receptors are activated in immature neurons, Cl^−^ flows out of the cell, making the membrane potential more positive. Therefore, in contrast to its effects on mature neurons, GABA can actually excite immature neurons (see figure 2) (Ben-Ari 2002). These excitatory actions of GABA during development contribute to its involvement in the control of neuronal growth, neuronal migration, and synapse formation/refinement. In rodents, the function of GABA_A_ receptors switches from excitatory to inhibitory at the end of the period equivalent to the third trimester (i.e., by postnatal days 10 to 12).

### Developmental Ethanol Exposure and GABA

Two recent studies highlight the importance of ethanol’s actions on the GABA neurotransmitter system during early developmental stages. The first study concerns the effect of ethanol on the generation of new neurons in the cerebral cortex. Sathyan and colleagues (2007) showed that fetal mouse cerebral cortical progenitor cells exposed to ethanol for 5 days (0.32 g/dl) decreased expression of small noncoding messenger RNA regulatory molecules (microRNAs), and the coordinated effects of ethanol on these microRNAs triggered premature maturation of the progenitor cells. The mechanism of action of ethanol involved, in part, activation of GABA_A_ receptors in the progenitor cells. The second study addressed the effect of ethanol on neuronal migration, demonstrating that exposing mice to a low concentration of ethanol in utero (see table 1) promoted premature migration of immature GABA interneurons into the cerebral cortex (Cuzon et al. 2008). Studies with cortical slices suggested that ethanol produces premature migration of immature GABAergic interneurons by increasing both ambient GABA levels and GABA_A_ receptor activation, which could act by stimulating these interneurons (see figure 2). These findings suggest that daily consumption of small amounts of ethanol (such as a glass of wine with meals) during the first and second trimesters of pregnancy could have significant effects on the development of GABAergic neurons in the fetus. Given the prominent role of GABA during development, this could significantly affect the normal development of cortical neuronal circuits. Collectively, these studies emphasize that ethanol can affect the function of the GABA neurotransmitter system even before synapses have been formed and that neurochemical imbalances can have profound consequences on early neuronal development.

During later stages of development, ethanol exposure also affects the maturation of GABAergic transmission. Hsiao and colleagues (2001) showed that administration of ethanol to rat pups via oral intubation during the third trimester–equivalent period (table 1) delays the developmental increase of GABA_A_ receptor–mediated currents in medial septum/diagonal band neurons, which are involved in modulation of attention, memory, and other cognitive functions. Because these processes are altered in FASD patients, future studies should investigate whether this is a consequence of deficits in the maturation of GABA input to these neurons.

Potent effects of ethanol exposure on GABA transmission during the third trimester–equivalent period also have been documented in the hippocampus—another brain region that is important for learning and memory processes. In a specific population of hippocampal neurons—namely, those located in the CA3 region—a primitive pattern of neuronal network oscillations has been well characterized. These oscillations are driven, in part, by the above-mentioned excitatory actions of GABA_A_ receptors (see figure 2). Galindo and colleagues (2005) demonstrated that acute ethanol exposure increases GABA release in the CA3 hippocampal region in brain slices from neonatal rats. This effect was produced by ethanol concentrations as low as 0.05 g/dl and ultimately results in an increase in neuronal oscillations. Whether ethanol also affects the switch in the actions of the GABA_A_ receptors from excitatory to inhibitory currently is under investigation.

Given that oscillatory network activity is thought to be important for the maturation of neuronal circuits in the hippocampus and other brain regions of several animal species including humans (Moody and Bosma 2005), it is possible that this effect of ethanol impairs the formation and/or refinement of synapses even when ethanol is consumed at low levels during late pregnancy. The oscillations control neuronal development by triggering changes in the activity of genes and/or inducing the release of trophic factors (from the Greek *trophe*, to nourish) that stabilize neuronal connections (Mohajerani and Cherubini 2006).

A recent study by Zucca and Valenzuela (2010) characterized the effect of ethanol on the release of trophic factors in the CA3 hippocampal region. Stimulation of pyramidal-shaped neurons in this region causes the dendritic release of a protein known as brain-derived neurotrophic factor (BDNF), which induces a long-lasting enhancement (i.e., potentiation) of GABA transmission that is thought to be essential for the maturation of GABA inputs to these neurons (Gaiarsa 2004). The researchers found that acute exposure to ethanol inhibits this BDNF-mediated potentiation of GABA transmission in hippocampal slices. This effect was very potent, reaching significance at concentrations as low as 0.025 g/dl, and was mediated by inhibition of the l-type category of voltage-gated calcium ion (Ca^2+^) channels that trigger the dendritic release of BDNF. This effect also was observed after repeated in vivo exposures to low doses of ethanol. Inhibition of this BDNF-dependent form of synaptic potentiation is likely to have a deleterious effect on the maturation of inhibitory circuits in the CA3 hippocampal region, causing an imbalance between excitatory and inhibitory synaptic transmission, and ultimately resulting in alterations in learning, memory, and other cognitive processes.

Developmental ethanol-induced alterations of GABA functioning also can produce long-lasting changes in neuronal circuits by inducing neuronal death. Exposure to high levels of ethanol (≥g/dl) during the third trimester–equivalent period was shown to cause widespread neuronal death in rats (Ikonomidou et al. 2000). This effect partially was mimicked by the administration of drugs such as barbiturates that enhance GABA_A_ receptor function and the researchers hypothesized that ethanol triggered cell death by inducing excessive inhibition of neuronal activity via GABA_A_ receptor potentiation. However, Sanderson and colleagues (2009) failed to demonstrate that ethanol directly enhances GABA_A_ receptor–mediated inhibition of cortical neurons in slices from neonatal rats, which is surprising given that these neurons were shown to be particularly sensitive to ethanol-induced cell death (Ikonomidou et al. 2000).

Persistent alterations in the GABA system could be responsible for behavioral abnormalities observed in the adult offspring from animals exposed to single or multiple doses of ethanol during development. However, the mechanisms responsible for these effects are presently unknown, including the possible connection between these persistent changes and any of the above-described effects of ethanol on the developing GABA neurotransmitter system. In one study of guinea pigs repeatedly exposed to high ethanol concentrations throughout pregnancy, researchers found persistent alterations in GABA transmission (see table 1) and increased levels of certain GABA_A_ receptor subunits (α_1_ and/or β_2/3_) in the cerebral cortex and hippocampus of adult offspring (Bailey et al. 2001). This effect could have occurred in response to reduced numbers of GABA neurons or a decrease in the enzymes essential to GABA synthesis (Bailey et al. 2001, 2004). Similarly, elevated levels of the α_5_ GABA_A_ receptor subunit were found in the brains of adult mice exposed to a single ethanol dose during gestational day 8 (neonatal ethanol levels expected to be near 0.5 g/dl) (Toso et al. 2006; Webster et al. 1983). GABA_A_ receptors containing the α_5_ subunit are expressed outside the synaptic area (see figure 1) and exert a persistent inhibitory control on neurons in the hippocampus and other brain regions, including the cerebral cortex (Pirker et al. 2000).

**Figure 3:**
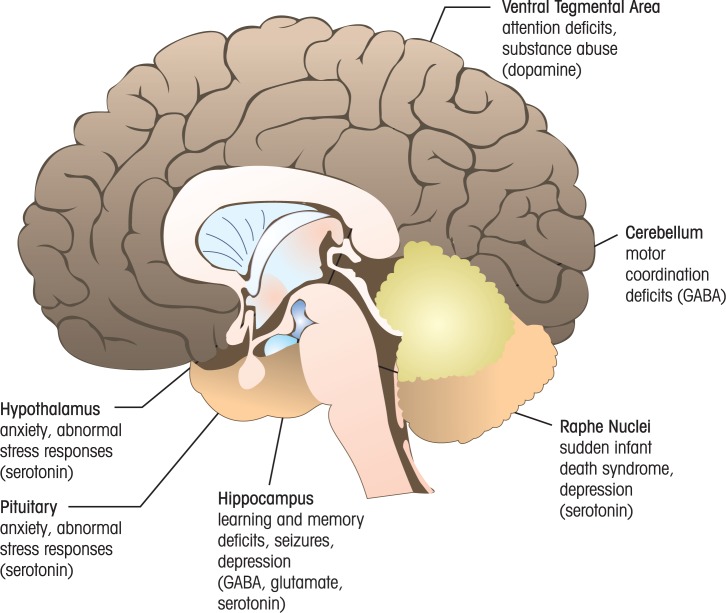
Examples of brain regions where chemical neurotransmitter system alterations have been demonstrated in models of fetal alcohol spectrum disorders (FASD). Shown is a schematic representation of the mature human brain. The potential FASD-linked conditions that could be explained by neurotransmitter system alterations are given under the label for each region. Anxiety and abnormal stress responses (serotonin) apply both to hypothalamus and pituitary. Examples of neurotransmitters that could potentially be involved in these deficits are given in parenthesis.

A study of adolescent monkeys exposed to ethanol in utero found reduced numbers of GABA neurons in the cerebral cortex (see table 1) (Miller 2006). Persistent dysfunction and/or loss of GABA neurons could, in part, be responsible for the increased susceptibility to epilepsy that has been linked to FASD (Bell et al. 2010; Bonthius et al. 2001). In addition, alterations in the function of a subtype of GABA neuron (i.e., the Purkinje neuron) in the cerebellum, a brain region important for motor coordination (see figure 3), could contribute to motor deficits observed in FASD patients (Green 2004; Hsiao et al. 1999; Servais et al. 2007). Collectively, these studies indicate that the GABA neurotransmitter system is an important target of developmental ethanol exposure and that further investigation of the mechanisms of action of ethanol on this system is warranted.

## Glutamate

Glutamate is the main excitatory neurotransmitter in the mammalian brain and is locally synthesized from glucose. It binds to two classes of receptors: glutamate-gated ion channels and G protein–coupled receptors, which regulate a variety of intracellular signaling pathways via activation of G proteins (i.e., guanine nucleotide–binding proteins). There are three families of glutamate-gated ion channels, named for the compounds that were used to initially identify these channels: (1) the *N*-methyl-d-aspartate (NMDA) receptor, (2) the α-amino-3-hydroxyl-5-methyl-4-isoxazole-propionate (AMPA) receptor, and (3) the kainate receptor (AMPA and kainate receptors are collectively known as non-NMDA receptors). There also are three families of G protein–coupled glutamate receptors, and these are denoted as group I, II, and III metabotropic glutamate receptors (mGluR I–III), based on their functional properties and effects on neuronal function.

### Developmental Ethanol Exposure and the Glutamate Receptors

#### Short-Term Effects

A significant amount of research in recent years has focused on whether NMDA receptors in developing and mature neurons have the same sensitivity to ethanol. Interest in this issue was kindled, in part, by controversial findings that administration of NMDA receptor blockers during the third trimester–equivalent period triggers neuronal degeneration in many areas of the rat brain. As discussed above, drugs that potentiate GABA_A_ receptors and ethanol produced a similar effect (Ikonomidou et al. 2000. For a detailed discussion of these findings, see Sanderson et al. 2009.) Given that numerous studies have shown that ethanol inhibits NMDA receptor function in mature neurons, it was hypothesized that ethanol acted, in part, by also inhibiting these receptors in immature neurons throughout the brain. Evidence supporting this hypothesis was found in a study with CA1 hippocampal neurons in slices from neonatal rats, where acute exposure to ethanol concentrations (0.18 to 0.36 g/dl) that are near those required to trigger neuronal degeneration (≥0.2 g/dl) inhibited NMDA receptor–mediated synaptic responses (Puglia and Valenzuela 2010*a*). However, acute exposure to ethanol (0.32 g/dl) did not affect NMDA receptor–mediated currents in another neuronal population that was shown to be particularly sensitive to ethanol-induced cell death: neocortical layer II/III neurons from neonatal rats (Sanderson et al. 2009).

Mameli and colleagues (2005) also have reported finding no direct, acute inhibitory action of ethanol on NMDA receptors in another population of developing rat hippocampal neurons (CA3 pyramidal neurons). The study found that ethanol sensitivity in these receptors is gradually acquired after the third trimester–equivalent period has been completed. The researchers did report that ethanol directly inhibits AMPA receptors in these neurons during the third trimester–equivalent period and that this sensitivity gradually disappears as development progresses. NMDA and AMPA receptors have distinct subunit compositions during development and maturity, and this could explain the differences in ethanol sensitivity that were observed. In addition, this study found that ethanol can indirectly affect the function of both AMPA and NMDA receptors in developing neurons by decreasing glutamate release; this effect was detected during the third trimester–equivalent period and was mediated by inhibition of the N-type of Ca^2+^ channels that mediates glutamate release onto immature but not mature CA3 pyramidal neurons. Taken together, these findings suggest that if direct inhibition of NMDA receptors by ethanol damages developing neurons, this mechanism cannot be generalized to all brain regions.

An alternative possibility is that inhibition of NMDA receptors during ethanol exposure does not injure developing neurons but that damage actually occurs during ethanol withdrawal and that compensatory increases in NMDA receptor levels and/or function result in excessive ion flux through this channel, which is known to trigger neuronal toxicity. This model is supported by independent studies in which ethanol-induced long-lasting learning deficits were shown to be prevented by administration of NMDA receptor antagonists during withdrawal from exposure to high ethanol levels (see table 1) in models of third trimester–equivalent, binge-like ethanol consumption (Lewis et al. 2007; Thomas et al. 2004).

As mentioned above with respect to GABA, synaptic potentiation mechanisms play a central role in synapse stabilization during the third trimester–equivalent period, and this also applies to glutamatergic synapses (Hanse et al. 2009). Researchers have hypothesized that ethanol could alter the maturation of glutamatergic circuits by interfering with synaptic potentiation mechanisms (Medina and Krahe 2008). Initial studies (Mameli and Valenzuela 2006; Valenzuela et al. 2008) focused on the early portion of the third trimester–equivalent period found that acute exposure to ethanol caused long-lasting potentiation of AMPA receptor-mediated transmission in the CA1 hippocampal region of neonatal rats via the local production and/or release of a steroid-like molecule. However, this potentiating effect of ethanol was not observed under all conditions; acute ethanol exposure was subsequently found to inhibit CA1 AMPA receptor–mediated responses using a different experimental approach (Puglia and Valenzuela 2009). Moreover, this study also found that repeated in vivo ethanol exposure did not affect glutamatergic transmission in the CA1 region. Further research examined this issue during the late portion of the third trimester–equivalent period; acute exposure to high concentrations of ethanol (0.36 g/dl) impaired synaptic potentiation in the CA1 region, indicating that the mechanism of action of acute ethanol exposure involves, at least in part, inhibition of the function of both NMDA receptor– and AMPA receptor–mediated synaptic responses (Puglia and Valenzuela 2010*a*). Importantly, glutamatergic synaptic potentiation also was impaired in neonatal rats repeatedly exposed to high ethanol levels during the third trimester–equivalent period (see table 1) (Puglia and Valenzuela, 2010*b*). Interestingly, in this in vivo study, neither AMPA nor NMDA receptor function was affected by repeated ethanol exposure, suggesting that acute and chronic ethanol exposure impair synaptic potentiation via different mechanisms. Collectively, these studies indicate that synaptic potentiation mechanisms in glutamatergic neuronal circuits are important targets of the short- and long-term actions of developmental ethanol exposure. Future studies should examine whether ethanol-induced impairments of these mechanisms alter maturation of hippocampal circuits and contribute to the long-lasting learning and memory deficits associated with FASD.

#### Long-Term Effects

In comparison to the action of ethanol on glutamatergic receptor function during development, more studies have examined the long-lasting effects of ethanol on NMDA receptors because they are involved in cellular mechanisms that are thought to be important for learning and memory. NMDA receptor–dependent long-term potentiation of AMPA receptor–mediated synaptic responses may underlie these processes and has been shown to be impaired in FASD (reviewed in Berman and Hannigan 2000). The NMDA receptor typically is formed by two NR1 subunits and two NR2 subunits. A number of studies carried out between 1988 and 1999 (reviewed in Costa et al. 2000) demonstrated that developmental ethanol exposure produces long-term changes in NMDA receptor levels in several brain regions. In the past decade, antibodies that selectively recognize specific NMDA receptor subunits have become widely available, and these have been used in several laboratories to further investigate the developmental effects of ethanol on NMDA receptor expression. These studies have yielded complex results that depend on the method of ethanol administration, dose of ethanol, brain region examined, experimental technique used to measure subunit levels, animal species, and animal age. Researchers have found increases, decreases, and no change in NMDA and/or AMPA receptor subunit levels (for examples see Dettmer et al. 2003; Honse et al. 2003; Naassila and Daoust 2002; Samudio-Ruiz et al. 2010). Long-lasting increases in NMDA receptor levels, coupled with elevated glucocorticoid and glutamate levels, could cause hippocampal damage, as suggested by a study with near-term fetal guinea pigs that were exposed throughout pregnancy to a high concentration of ethanol (see table 1) (Iqbal et al. 2006).

Long-lasting potentiation and other forms of synaptic plasticity (including long-term depression, the counterpart of long-lasting potentiation) can be impaired by certain patterns of developmental ethanol exposure, and this also could be a consequence of persistent alterations in NMDA receptor levels and/or function, including deficits in activation of NMDA receptor–dependent intracellular signaling pathways (Izumi et al. 2005; Margret et al. 2006; Medina and Krahe 2008; Richardson et al. 2002). For instance, NMDA receptor–dependent activation of extracellular receptor–activated kinase, a key enzyme that relays signals from the cell membrane to the nucleus during long-term synaptic potentiation, recently was shown to be impaired in adult mice exposed throughout pregnancy to relatively low levels of ethanol (see table 1) (Samudio-Ruiz et al. 2009). The mechanisms responsible for these long-lasting effects of developmental ethanol exposure presently are unknown—including whether there is a connection between these and the developmental actions of ethanol on glutamatergic transmission—and none of these studies has conclusively linked these NMDA receptor alterations with behavioral deficits in animal models of FASD.

Long-lasting changes in AMPA receptor function also have been detected in a few recent studies. Increases in AMPA receptor function were detected in medial septum/diagonal band neurons from juvenile rats exposed to ethanol in a binge-like fashion during the third trimester–equivalent period (see table 1) (Hsiao and Frye 2003). Decreases in frequency and amplitude of spontaneous AMPA receptor–mediated currents were found in CA1 hippocampal pyramidal neurons from 18- to 27-day-old rats exposed to ethanol in utero, and this effect was ameliorated by postnatal administration of an agent that stimulates AMPA receptor function (i.e., aniracetam) (see table 1) (Wijayawardhane et al. 2007, 2008). Importantly, this aniracetam treatment regimen reversed learning and memory deficits that were present in untreated rats when they reached 40 days of age (Vaglenova et al. 2008). Clearly, further research efforts should focus on the role of AMPA receptors in the learning disabilities associated with FASD.

Metabotropic GluRs are powerful modulators of synaptic transmission in many brain regions, including the regulation of synaptic potentiation. Metabotropic GluRs have been implicated in learning and memory processes, as well as a human intellectual disabilities (Fragile X syndrome), where the absence of a protein encoded by the Fragile X mental retardation 1 (FMR1) gene causes dysregulation of mGluR-dependent signalling (Bassell and Warren 2008). Metabotropic GluRs also are known to regulate proliferation, differentiation, and survival of neuronal progenitor cells (Catania et al. 2007). Despite the importance of mGluRs for neuronal development, only a handful of studies have examined their role in FASD, and these have only focused on the long-term effects of ethanol. Queen and colleagues (1993) found a reduction in mGluR function in the hippocampus of adult offspring from rats exposed to ethanol during pregnancy (at a blood ethanol level of 0.08 g/dl). Similar findings were reported in another study with rats exposed to ethanol during gestational days 12 to 20 (blood ethanol levels not reported) (Noble and Ritchie 1989). In contrast, Valles and colleagues (1995) found that when rat dams were exposed to ethanol throughout gestation (maternal blood ethanol levels ∼0.1 g/dl) as well as during the lactation period (neonatal blood ethanol levels reported as very low), there was an increase in mGluR function in the hippocampus of juvenile offspring. More recently, Galindo and colleagues (2004) showed that levels and function of a specific mGluR subunit (mGluR5) were decreased in part of the hippocampus (i.e., dentate gyrus) of adult offspring of rats gestationally exposed to ethanol (see table 1). In another study, the long-term effects of ethanol were assessed in cultured cerebellar neurons obtained from embryonic day 20 rat fetuses. Exposure of these neurons to 0.15 g/dl of ethanol for 9 to 11 days decreased mGluR function; however, upon 1 day of withdrawal, these responses were enhanced (Netzeband et al. 2002). These studies indicate that mGluRs are important targets of ethanol exposure during development and future research should investigate the role of these receptors in the behavioral abnormalities associated with FASD.

## Serotonin

This neurotransmitter, also known as 5-hydroxytryptamine (5-HT), is synthesized from tryptophan by the enzymes tryptophan hydroxylase and aromatic amino acid decarboxylase. It binds to two types of receptors: serotonin-gated ion channels (5-HT_3_ receptors) or G protein–coupled receptors (5-HT_1_, 5-HT_2_, and 5-HT_4–7_ receptors). The body of most serotonin neurons is located in a series of nuclei—known as the raphe nuclei—that are shaped like a seam and are located in the brain stem (see figure 3). Some serotonergic projections from these nuclei descend to the spinal cord where they modulate pain transmission. Other projections ascend to brain regions such as the cortex, hippocampus, and hypothalamus (see figure 3). In the mature brain, the serotonin neurotransmitter system is involved in the regulation of mood, attention, appetite, sleep, and other functions. Alterations in this neurotransmitter system have been linked to neuropsychiatric conditions, including depression. Serotonin is removed from synapses via reuptake mediated by transporters in the axon terminals, which are inhibited by antidepressant medications such as fluoxetine (Prozac^®^). Serotonin neurons are expressed early in development (embryonic day 12 in rodents), and serotonin released from these neurons has been shown to control progenitor cell proliferation, differentiation, migration, and synapse formation (Frederick and Stanwood 2009). Therefore, the role of alterations in this transmitter system in FASD has been investigated in several laboratories, as these alterations could have a wide impact on neuronal circuit development across different brain regions.

### Developmental Ethanol Exposure and Serotonin

Goodlett and colleagues (2005) recently reviewed the effects of developmental ethanol exposure on the serotonergic neurotransmitter system, and the reader is referred to this article for more details. Briefly, studies have shown that developmental ethanol exposure, particularly when it involves exposure during the first and/or second trimester equivalents, decreases differentiation, migration, and axonal outgrowth of serotonin neurons. The mechanism underlying these effects may involve alterations in crosstalk between glial and neuronal cells. Serotonin neurons release serotonin onto glial cells, activating 5-HT_1A_ receptors expressed in these cells and inducing them to release neurotrophic factors (for instance, a protein known as S100P) that feed-back onto serotonin neurons to nurture them. Activators of 5-HT_1A_ receptors and some neuroprotective peptides have been shown to ameliorate the effects of ethanol on serotonin neuron development (Druse et al. 2005; Zhou et al. 2008).

Several recent studies have investigated the impact of serotonin deficits induced by developmental ethanol exposure. Zhou and colleagues (2005) found that decreased serotonin innervation correlated with reduced numbers of thalamocortical fibers, as well as decreased size of regions targeted by serotonin axons, including the hypothalamus, cerebral cortex, and hippocampus (see figure 3). Kinney and colleagues (2003) discovered a link among deficits in the brain stem serotonin system, prenatal ethanol exposure, prenatal nicotine exposure, and sudden infant death syndrome in autopsy cases from Native American Indians from the Northern Plains. Prenatal ethanol exposure is a major risk factor for sudden infant death syndrome, and this may be a consequence of ethanol–induced abnormalities in the maturation of serotonin neurons in the brain stem, where these neurons play an important role in the control of respiration, heart function, and blood pressure. In agreement with these human studies, developmental ethanol exposure in rats was shown to alter respiratory long-term facilitation, a serotonin-dependent protective mechanism that takes place in brain stem neurons in response to repeated events of low oxygenation (Kervern et al. 2009). In animals that are chronically exposed to ethanol, low oxygenation paradoxically induced long-term depression in these neurons in response to low oxygenation. Application of a 1-μM concentration of the serotonin analog, α-methyl-serotonin, induced respiratory long-term facilitation and depression in control and ethanol groups, respectively. These data are consistent with the model that prenatal ethanol exposure-induced alterations on serotonin modulation of brain stem neurons that control respiration could explain the high incidence of sudden infant death syndrome in FASD. Hofmann and colleagues (2007) observed complex gender-dependent effects on serotonergic modulation of the hypothalamic–pituitary–adrenal axis in adult rat offspring repeatedly exposed to ethanol during prenatal development. This axis is part of the neuroendocrine system and controls stress responses among other important physiological processes (see figure 3). Moreover, Kraemer and colleagues (2008) reported evidence suggesting an interesting interaction between repeated prenatal ethanol exposures and a variation (DNA polymorphism) in the serotonin transporter gene. They found that prenatal ethanol exposed monkeys carrying this serotonin gene variation were more irritable as neonates and exhibited increased stress hormone levels. For more details on the role of serotonin and other neurotransmitters in alterations of stress responses by developmental ethanol exposure, which can be responsible for many of the alterations associated with FASD, including depression, anxiety, learning and memory deficits, and increased susceptibility to infections, see Lee and colleagues (2008) and Weinberg and colleagues (2008).

As mentioned above, alterations in the serotonin neurotransmitter system play a role in depression, and this mood disorder often is present in FASD patients (O’Connor and Paley 2006). Depressive-like behavior also has been detected in rodent models of FASD, and this could be a consequence of reductions in levels of BDNF (see GABA section above) (Caldwell et al. 2008; Castrén and Rantamäki 2008). Antidepressant medications that act by inhibiting serotonin uptake have been shown to restore BDNF levels, suggesting that serotonin controls BDNF production by neurons (Martinowich and Lu 2008). It is therefore possible that deficits in serotonin transmission induced by developmental ethanol exposure result in long-lasting changes in BDNF levels and that this is, in part, responsible for the increased incidence of depression in FASD patients. Future studies should test this possibility.

## Dopamine

This biogenic amine transmitter is synthesized from tyrosine by the enzymes tyrosine hydroxylase and l-aromatic amino acid decarboxylase. Dopamine binds to five types of G protein–coupled receptors that are grouped in two families: D_1_-like receptors (D_1_ and D_5_ receptors) and D_2_-like receptors (D_2_, D_3_, and D_4_ receptors). The action of dopamine is terminated by reuptake into axonal terminals via dopamine transporters. The cell bodies of dopaminergic neurons are located in the hypothalamus and in brain stem regions known as the substantia nigra pars compacta and the ventral tegmental area (see figure 3). Dopaminergic fibers project extensively throughout many brain regions, including the cortex, hippocampus, and striatum. In the mature brain, dopamine is involved in the regulation of movement, attention, motivation, and reward. Alterations in this neurotransmitter system have been linked to neurological disorders, such as Parkinson’s disease (caused by degeneration of substantia nigra pars compacta dopaminergic neurons), as well as neuropsychiatric conditions such as schizophrenia, attention deficit disorder, and substance abuse. In the developing brain, dopamine regulates neuronal differentiation, migration (including that of GABAergic neurons), and axonal and/or dendritic growth (Frederick and Stanwood 2009).

### Developmental Ethanol Exposure and Dopamine

Although it is well established that the dopaminergic system is an important target of the developmental actions of substances of abuse and environmental toxins (Frederick and Stanwood 2009; Thompson et al. 2009), comparatively little is known about the actions of ethanol on this neurotransmitter system.

FASD is associated with attention deficits and increased susceptibility to substance abuse, and this could be the result of alterations in the dopaminergic neurotransmitter system. Studies carried out in the 1980s and 1990s demonstrated that repeated prenatal ethanol exposure decreases dopamine content and turnover, reduces D1 receptor levels, distorts the shape of dopaminergic neurons, and affects sensitivity of dopamine receptors to pharmacological agents (see Wang et al. 2006 and references therein). More recently, a positron emission tomography study detected complex alterations in the dopaminergic neurotransmitter system in young adult Rhesus monkeys that were exposed to low levels of ethanol during different phases of pregnancy (see table 1) (Schneider et al. 2005). Other studies (see Wang et al. 2006 and references therein) have demonstrated that prenatal ethanol exposure persistently reduces the number of spontaneously active dopamine neurons in the ventral tegmental area and substantia nigra of developing and adult offspring (see table 1). The mechanism by which ethanol produces this effect is unknown, but it appears to involve inactivation of a population of dopaminergic neurons in these regions. Importantly, methyphenidate (Ritalin^®^), a drug used to treat attention deficit disorders, restores normal dopaminergic neuronal activity for a prolonged period of time in rats exposed to ethanol prenatally (Shen and Choong 2006). The precise mechanism by which methylphenidate produces this effect is presently unknown.

In addition to mediating FASD-related attention deficits, alterations in the dopaminergic neurotransmitter system also could underlie the increased incidence of ethanol abuse and other substance abuse disorders that has been observed in individuals exposed to ethanol in utero as well as in animal models of FASD (Alati et al. 2006; Baer et al. 2003; Matta and Elberger 2007; Yates et al. 1998). Barbier and colleagues (2008, 2009) found that exposure of rats to ethanol during pregnancy and lactation increased ethanol consumption and sensitivity to its anxiety-decreasing effects in adult offspring (see table 1). These animals also exhibited increased sensitivity to the rewarding effects of cocaine and increased sensitization to cocaine and amphetamine (these drugs act in part by increasing levels of dopamine in the synapses). Decreases in D_1_ and/or dopamine transporter levels were detected in the striatum of these rats. Youngentob and Glendinning (2009) recently reported that ethanol tasted and smelled better to the adolescent offspring of rats exposed to ethanol during pregnancy, resulting in increased ethanol intake. Although the mechanism of this effect is not fully understood, it may involve alterations in the levels of neurotransmitter genes in the olfactory bulb, including levels of the D_2_ receptor gene (Middleton et al. 2009). Future studies will be needed to determine if there is a link between these alterations in D_2_ receptor expression and changes in olfactory ethanol sensitivity.

## Conclusion

**Figure 4:**
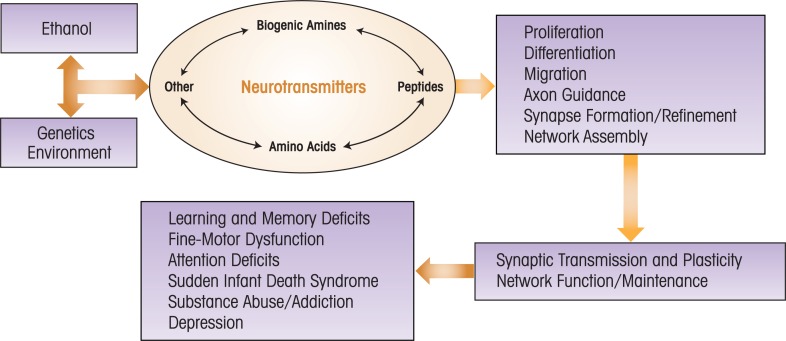
Diagram illustrating the potential role of neurotransmitter system alterations in fetal alcohol spectrum disorders (FASD). Ethanol exposure during development, acting in conjunction with genetic susceptibility factors (for instance, variations in the serotonin transporter gene) and environmental factors (for example, coexposure to nicotine), disrupts the actions of neurotransmitter systems (i.e., biogenic amines, etc) that normally interact in a complex manner to regulate the key processes involved in brain development (i.e. proliferation, etc). Disruption of these processes results in persistent alterations in synaptic transmission/plasticity and neuronal network function. These alterations likely underlie the deficits associated with FASD (i.e., learning and memory deficits). The precise chain of events leading from developmental ethanol exposure to these deficits remains to be determined.

Research on the effects of developmental ethanol exposure on chemical neurotransmitter systems has significantly increased over the past decade. Studies have convincingly demonstrated that neurotransmission in the developing brain does not always respond to ethanol as in the adult brain and that components of developing neurotransmitter systems have unique properties that make them particularly sensitive to the adverse actions of ethanol, even at low levels of exposure. Ethanol-induced abnormalities in the formation and refinement of developing neuronal circuits are likely to be, in part, responsible for the persistent structural and functional brain deficits that characterize FASD. These deficits are ultimately responsible for the behavioral and cognitive alterations present in patients with this disorder and for their increased propensity to have comorbid neuropsychiatric diseases and some neurolgical disorders (see figures 3 and 4). Future studies should continue to investigate the mechanisms by which ethanol affects amino acid and biogenic amine neurotransmitter systems, and extend this work to other neurotransmitter systems, including peptide neurotransmitters. It is important to also continue to investigate the effects of developmental ethanol exposure on modulators of neurotransmission, as these may be key targets for the development of effective therapeutic interventions against FASD.

## Figures and Tables

**Table 1:** Examples of Recent Studies on the Effects of Developmental Ethanol Exposure on Neurotransmitter Systems*

**Neurotransmitter System**	**Effect of Developmental Exposure**	**Exposure Period†**	**Mode of Ethanol Administration**	**Blood Ethanol Levels (g/dl)‡**	**Species**	**Reference**
γ–Aminobutyric acid (GABA)	Premature migration of cortical GABAergic interneurons at E14.5	E0.5–E14.5	Liquid diet	0.025 (maternal)	Mice	(Cuzon et al. 2008)
	Altered plasticity and firing of cerebellar GABAergic neurons (Purkinje) at P15–P20	E0–E21	Drinking water	0.08 (maternal)	Mice	(Servais et al. 2007)
	Decrease in levels of GABA_A_ receptor α5 subunit at E18 and increased expression in adults	E8	Intraperitoneal injection	Not determined	Mice	(Toso et al. 2006)
	Increase in levels of GABA_A_ receptor α_1_ and β_2/3_ subunit in adults	E2–E67	Oral intubation	0.32 (maternal)	Guinea Pigs	(Bailey et al. 2001)
	Decrease in cortical GABAergic (and glutamatergic) neuronal numbers during adolescence	E3–E42/168	Intragastric intubation	0.23 (maternal)	Monkeys	(Miller 2006)
	Impaired brain-derived neurotrophic factor (BDNF)-dependent plasticity of hippocampal GABAergic transmission at P4–P6	P2–P6	Vapor chambers	0.025–0.18 (neonate)	Rats	(Zucca and Valenzuela 2010)
	Delayed GABAergic current maturation in medial septum/diagonal band neurons at P12–P15	P4–P6	Oral intubation	0.28 (neonate)	Rats	(Hsiao et al. 2001)
	Widespread neuronal death at P8 potentially caused by ethanol-induced enhancement of GABA_A_ receptors (and inhibition of *N*-methyl-d-aspartic acid [NMDA] receptors)	P7	Subcutaneous injection	≥0.2 (neonate)	Rats	(Ikonomidou et al. 2000)
Glutamate	Increase in hippocampal glutamate and NMDA receptor levels at E63	E2–E63	Oral intubation	0.28 (maternal)	Guinea Pigs	(Iqbal et al. 2006)
	Impaired glutamatergic transmission and plasticity in the hippocampus	E2–E67	Oral intubation	0.28 (maternal)	Guinea Pigs	(Richardson et al. 2002)
	Impaired NMDA receptor–dependent activation of extracellular receptor–activated kinase	E0–E21	Voluntary drinking using two bottle choice paradigm	g 0.08 (maternal)	Mice	(Samudio-Ruiz et al. 2009)
	Decrease in NR2A and NR2B NMDA subunit mRNA in the hippocampus. Increase in NR2A mRNA in the cortex and NR2B in cortex and cerebellum.	E8	Intraperitoneal injection	Not determined	Mice	(Incerti et al. 2010)
	Learning or motor deficits in adult animals that could be prevented by NMDA receptor antagonism during withdrawal	P6 (learning) P1–P8 (motor	) Gastric intubation	0.3–0.4 (learning) 0.22 (motor) (neonate)	Rats	(Lewis et al. 2007; Thomas et al. 2004)
	Impaired hippocampal glutamatergic plasticity at P7–P9	P2–P9	Vapor chambers	0.3–0.4 (neonate)	Rats	(Valenzuela 2010*b*)
	Impaired hippocampal plasticity at P30	P9	Subcutaneous injection	0.2–0.5 (neonate)	Rats	(Izumi etal. 2005)
	Decrease in AMP-activated protein kinase (AMPA) receptor currents in the hippocampus at P18–P27	E3–E20	Intragastric intubation	0.18 (maternal)	Rats	(Wijayawardhane et al. 2007)
	Increase in AMPA receptor function in medial septum/diagonal band neurons at P32–P35	P4–P9	Oral intubation	0.35 (neonate)	Rats	(Hsiao and Frye 2003)
	Decrease in levels and function of mGluR5 in the dentate gyrus of adult animals	E3–E21	Liquid diet	0.07–0.14 (maternal)	Rats	(Galindo etal. 2004)
Serotonin	Decreased serotonin innervation correlated with decreased size in regions targeted by this transmitter at E15–E18	E7–E15/18	Liquid diet	0.07–0.14 (maternal)	Rats	(Zhou et al. 2005)
	Increased incidence in sudden infant death syndrome that correlated with serotonergic abnormalities in the brain stem at 40–90 postconceptional weeks	Unknown	Oral ingestion	Unknown		(Kinney et al. Humans 2003)
	Impaired serotonin-dependent respiratory long-term facilitation of brain stem neurons at P5–P7	E0–E21	Drinking water	0.08 (maternal)	Rats	(Kervern et al. 2009)
	Alterations in serotonergic modulation of hypothalamic–pituitary–adrenal axis	E1–E21	Liquid diet	Not determined	Rats	(Hofmann et al. 2007)
	Presence of a serotonin transporter DNA sequence variation (polymorphism) was associated with increased irritability and stress hormone levels during the neonatal period in animals exposed to ethanol in utero.	E0–E164	Oral ingestion	0.02–0.05 (maternal)	Monkeys	(Kraemer et al. 2008)
Dopamine	Persistent reduction in number of spontaneously active dopaminergic neurons in the ventral tegmental area and substantia nigra of developing and adult offspring	E8–E20	Gastric intubation	0.3 (maternal)	Rats	(Wang et al. 2006)
	Decrease in D1 receptor and dopamine transporter levels	E0–E21 plus lactation	Drinking water and mother’s milk	0.08 (maternal)§	Rats	(Barbier et al. 2008)
	Early-gestation ethanol exposure reduced dopaminergic function in adulthood. Middle- to late-gestation exposure heightened dopaminergic function	E0–E50, E50–E135, or E0–E135	Oral ingestion	0.02–0.05 (maternal)	Monkeys	(Schneider et al. 2005)

NOTE:

* Only recent studies that used in vivo ethanol exposure paradigms were included. See text for discussion of in vitro studies on the acute effects of ethanol.

† Duration of pregnancy is approximately 21 days in rats and mice, 68 days in guinea pigs, 160–180 days in monkeys, and 280 days in humans. E, embryonic; P, postnatal

‡ Legal intoxication limit in the U.S. = 0.08 g/dl.

§ Levels in nursing pups were not measured but are expected to be significantly lower than maternal levels.

## Footnotes

1 The terms ethanol and alcohol are used interchangeably in this article.

## References

[b1-arh-34-1-106] Alati R, Al Mamun A, Williams GM (2006). In utero alcohol exposure and prediction of alcohol disorders in early adulthood: A birth cohort study. Archives of General Psychiatry.

[b2-arh-34-1-106] Baer JS, Sampson PD, Barr HM (2003). A 21-year longitudinal analysis of the effects of prenatal alcohol exposure on young adult drinking. Archives of General Psychiatry.

[b3-arh-34-1-106] Bailey CD, Brien JF, Reynolds JN (2001). Chronic prenatal ethanol exposure increases GABA(A) receptor subunit protein expression in the adult guinea pig cerebral cortex. Journal of Neuroscience.

[b4-arh-34-1-106] Bailey CD, Brien JF, Reynolds JN (2004). Chronic prenatal ethanol exposure alters the proportion of GABAergic neurons in layers II/III of the adult guinea pig somatosensory cortex. Neurotoxicology and Teratology.

[b5-arh-34-1-106] Barbier E, Houchi H, Warnault V (2009). Effects of prenatal and postnatal maternal ethanol on offspring response to alcohol and psychostimulants in Long Evans rats. Neuroscience.

[b6-arh-34-1-106] Barbier E, Pierrefiche O, Vaudry D (2008). Long-term alterations in vulnerability to addiction to drugs of abuse and in brain gene expression after early life ethanol exposure. Neuropharmacology.

[b7-arh-34-1-106] Bassell GJ, Warren ST (2008). Fragile X syndrome: Loss of local mRNA regulation alters synaptic development and function. Neuron.

[b8-arh-34-1-106] Bell SH, Stade B, Reynolds JN (2010). The remarkably high prevalence of epilepsy and seizure history in fetal alcohol spectrum disorders. Alcoholism: Clinical and Experimental Research.

[b9-arh-34-1-106] Ben-Ari Y (2002). Excitatory actions of GABA during development: The nature of the nurture. Nature Reviews Neuroscience.

[b10-arh-34-1-106] Ben-Ari Y (2008). Neuro-archaeology: Pre-symptomatic architecture and signature of neurological disorders. Trends in Neurosciences.

[b11-arh-34-1-106] Berman RF, Hannigan JH (2000). Effects of prenatal alcohol exposure on the hippocampus: Spatial behavior, electrophysiology, and neuroanatomy. Hippocampus.

[b12-arh-34-1-106] Bonthius DJ, Pantazis NJ, Karacay B (2001). Alcohol exposure during the brain growth spurt promotes hippocampal seizures, rapid kindling, and spreading depression. Alcoholism: Clinical and Experimental Research.

[b13-arh-34-1-106] Caldwell KK, Sheema S, Paz RD (2008). Fetal alcohol spectrum disorder-associated depression: Evidence for reductions in the levels of brain-derived neurotrophic factor in a mouse model. Pharmacology, Biochemistry, and Behavior.

[b14-arh-34-1-106] Castrén E, Rantamäki T (2008). Neurotrophins in depression and antidepressant effects. Novartis Foundation Symposium.

[b15-arh-34-1-106] Catania MV, D’Antoni S, Bonaccorso CM (2007). Group I metabotropic glutamate receptors: A role in neurodevelopmental disorders?. Molecular Neurobiology.

[b16-arh-34-1-106] Cline H, Haas K (2008). The regulation of dendritic arbor development and plasticity by glutamatergic synaptic input: A review of the synaptotrophic hypothesis. Journal of Physiology.

[b17-arh-34-1-106] Costa ET, Savage DD, Valenzuela CF (2000). A review of the effects of prenatal or early postnatal ethanol exposure on brain ligand-gated ion channels. Alcoholism: Clinical and Experimental Research.

[b18-arh-34-1-106] Cuzon VC, Yeh PW, Yanagawa Y (2008). Ethanol consumption during early pregnancy alters the disposition of tangentially migrating GABAergic interneurons in the fetal cortex. Journal of Neuroscience.

[b19-arh-34-1-106] Dettmer TS, Barnes A, Iqbal U (2003). Chronic prenatal ethanol exposure alters ionotropic glutamate receptor subunit protein levels in the adult guinea pig cerebral cortex. Alcoholism: Clinical and Experimental Research.

[b20-arh-34-1-106] Druse M, Tajuddin NF, Gillespie RA, Le P (2005). Signaling pathways involved with serotonin1A agonist-mediated neuroprotection against ethanol-induced apoptosis of fetal rhombencephalic neurons. Brain Research Developmental Brain Research.

[b21-arh-34-1-106] Frederick AL, Stanwood GD (2009). Drugs, biogenic amine targets and the developing brain. Developmental Neuroscience.

[b22-arh-34-1-106] Gaiarsa JL (2004). Plasticity of GABAergic synapses in the neonatal rat hippocampus. Journal of Cellular and Molecular Medicine.

[b23-arh-34-1-106] Galindo R, Frausto S, Wolff C (2004). Prenatal ethanol exposure reduces mGluR5 receptor number and function in the dentate gyrus of adult offspring. Alcoholism: Clinical and Experimental Research.

[b24-arh-34-1-106] Galindo R, Zamudio PA, Valenzuela CF (2005). Alcohol is a potent stimulant of immature neuronal networks: Implications for fetal alcohol spectrum disorder. Journal of Neurochemistry.

[b25-arh-34-1-106] Goodlett CR, Horn KH, Zhou FC (2005). Alcohol teratogenesis: Mechanisms of damage and strategies for intervention. Experimental Biology and Medicine (Maywood).

[b26-arh-34-1-106] Green JT (2004). The effects of ethanol on the developing cerebellum and eyeblink classical conditioning. Cerebellum.

[b27-arh-34-1-106] Hanse E, Taira T, Lauri S, Groc L (2009). Glutamate synapse in developing brain: An integrative perspective beyond the silent state. Trends in Neurosciences.

[b28-arh-34-1-106] Hofmann CE, Ellis L, Yu WK, Weinberg J (2007). Hypothalamic-pituitary-adrenal responses to 5-HT1A and 5-HT2A/C agonists are differentially altered in female and male rats prenatally exposed to ethanol. Alcoholism: Clinical and Experimental Research.

[b29-arh-34-1-106] Honse Y, Nixon KM, Browning MD, Leslie SW (2003). Cell surface expression of NR1 splice variants and NR2 subunits is modified by prenatal ethanol exposure. Neuroscience.

[b30-arh-34-1-106] Hsiao SH, Acevedo JL, DuBois DW (2001). Early postnatal ethanol intubation blunts GABA(A) receptor up-regulation and modifies 3alpha-hydroxy-5alpha-pregnan-20-one sensitivity in rat MS/DB neurons. Brain Research Developmental Brain Research.

[b31-arh-34-1-106] Hsiao SH, Frye GD (2003). AMPA receptors on developing medial septum/diagonal band neurons are sensitive to early postnatal binge-like ethanol exposure. Brain Research Developmental Brain Research.

[b32-arh-34-1-106] Hsiao SH, West JR, Mahoney JC, Frye GD (1999). Postnatal ethanol exposure blunts upregulation of GABAA receptor currents in Purkinje neurons. Brain Research.

[b33-arh-34-1-106] Ikonomidou C, Bittigau P, Ishimaru MJ (2000). Ethanol-induced apoptotic neurodegeneration and fetal alcohol syndrome. Science.

[b34-arh-34-1-106] Incerti M, Vink J, Roberson R (2010). Reversal of alcohol-induced learning deficits in the young adult in a model of fetal alcohol syndrome. Obstetrics and Gynecology.

[b35-arh-34-1-106] Iqbal U, Brien JF, Kapoor A (2006). Chronic prenatal ethanol exposure increases glucocorticoid-induced glutamate release in the hippocampus of the near-term foetal guinea pig. Journal of Neuroendocrinology.

[b36-arh-34-1-106] Izumi Y, Kitabayashi R, Funatsu M (2005). A single day of ethanol exposure during development has persistent effects on bi-directional plasticity, N-methyl-D-aspartate receptor function and ethanol sensitivity. Neuroscience.

[b37-arh-34-1-106] Kervern M, Dubois C, Naassila M (2009). Perinatal alcohol exposure in rat induces long-term depression of respiration after episodic hypoxia. American Journal of Respiratory and Critical Care Medicine.

[b38-arh-34-1-106] Kinney HC, Randall LL, Sleeper LA (2003). Serotonergic brainstem abnormalities in Northern Plains Indians with the sudden infant death syndrome. Journal of Neuropathology and Experimental Neurology.

[b39-arh-34-1-106] Kraemer GW, Moore CF, Newman TK (2008). Moderate level fetal alcohol exposure and serotonin transporter gene promoter polymorphism affect neonatal temperament and limbic-hypothalamic-pituitary-adrenal axis regulation in monkeys. Biological Psychiatry.

[b40-arh-34-1-106] Lee S, Choi I, Kang S, Rivier C (2008). Role of various neurotransmitters in mediating the long-term endocrine consequences of prenatal alcohol exposure. Annals of the New York Academy of Sciences.

[b41-arh-34-1-106] Lewis B, Wellmann KA, Barron S (2007). Agmatine reduces balance deficits in a rat model of third trimester binge-like ethanol exposure. Pharmacology, Biochemistry, and Behavior.

[b42-arh-34-1-106] Mameli M, Valenzuela CF (2006). Alcohol increases efficacy of immature synapses in a neurosteroid-dependent manner. European Journal of Neuroscience.

[b43-arh-34-1-106] Mameli M, Zamudio PA, Carta M, Valenzuela CF (2005). Developmentally regulated actions of alcohol on hippocampal glutamatergic transmission. Journal of Neuroscience.

[b44-arh-34-1-106] Manent JB, Represa A (2007). Neurotransmitters and brain maturation: Early paracrine actions of GABA and glutamate modulate neuronal migration. Neuroscientist.

[b45-arh-34-1-106] Margret CP, Li CX, Chappell TD (2006). Prenatal alcohol exposure delays the development of the cortical barrel field in neonatal rats. Experimental Brain Research.

[b46-arh-34-1-106] Martinowich K, Lu B (2008). Interaction between BDNF and serotonin: Role in mood disorders. Neuropsychopharmacology.

[b47-arh-34-1-106] Matta SG, Elberger AJ (2007). Combined exposure to nicotine and ethanol throughout full gestation results in enhanced acquisition of nicotine self-administration in young adult rat offspring. Psychopharmacology (Berl).

[b48-arh-34-1-106] Medina AE, Krahe TE (2008). Neocortical plasticity deficits in fetal alcohol spectrum disorders: Lessons from barrel and visual cortex. Journal of Neuroscience Research.

[b49-arh-34-1-106] Middleton FA, Carrierfenster K, Mooney SM, Youngentob SL (2009). Gestational ethanol exposure alters the behavioral response to ethanol odor and the expression of neurotransmission genes in the olfactory bulb of adolescent rats. Brain Research.

[b50-arh-34-1-106] Miller MW (2006). Effect of prenatal exposure to ethanol on glutamate and GABA immunoreactivity in macaque somatosensory and motor cortices: Critical timing of exposure. Neuroscience.

[b51-arh-34-1-106] Mohajerani MH, Cherubini E (2006). Role of giant depolarizing potentials in shaping synaptic currents in the developing hippocampus. Critical Reviews in Neurobiology.

[b52-arh-34-1-106] Moody WJ, Bosma MM (2005). Ion channel development, spontaneous activity, and activity-dependent development in nerve and muscle cells. Physiology Reviews.

[b53-arh-34-1-106] Naassila M, Daoust M (2002). Effect of prenatal and postnatal ethanol exposure on the developmental profile of mRNAs encoding NMDA receptor subunits in rat hippocampus. Journal of Neurochemistry.

[b54-arh-34-1-106] Netzeband JG, Schneeloch JR, Trotter C (2002). Chronic ethanol treatment and withdrawal alter ACPD-evoked calcium signals in developing Purkinje neurons. Alcoholism: Clinical and Experimental Research.

[b55-arh-34-1-106] Noble EP, Ritchie T (1989). Prenatal ethanol exposure reduces the effects of excitatory amino acids in the rat hippocampus. Life Sciences.

[b56-arh-34-1-106] O’Connor MJ, Paley B (2006). The relationship of prenatal alcohol exposure and the postnatal environment to child depressive symptoms. Journal of Pediatric Psychology.

[b57-arh-34-1-106] Pirker S, Schwarzer C, Wieselthaler A (2000). GABA(A) receptors: Immunocytochemical distribution of 13 subunits in the adult rat brain. Neuroscience.

[b58-arh-34-1-106] Puglia MP, Valenzuela CF (2009). AMPAR-mediated synaptic transmission in the CA1 hippocampal region of neonatal rats: Unexpected resistance to repeated ethanol exposure. Alcohol.

[b59-arh-34-1-106] Puglia MP, Valenzuela CF (2010a). Ethanol acutely inhibits ionotropic glutamate receptor-mediated responses and long-term potentiation in the developing CA1 hippocampus. Alcoholism: Clinical and Experimental Research.

[b60-arh-34-1-106] Puglia MP, Valenzuela CF (2010b). Repeated third trimester-equivalent ethanol exposure inhibits long-term potentiation in the hippocampal CA1 region of neonatal rats. Alcohol.

[b61-arh-34-1-106] Queen SA, Sanchez CF, Lopez SR (1993). Dose- and age-dependent effects of prenatal ethanol exposure on hippocampal metabotropic-glutamate receptor-stimulated phosphoinositide hydrolysis. Alcoholism: Clinical and Experimental Research.

[b62-arh-34-1-106] Ramocki MB, Zoghbi HY (2008). Failure of neuronal homeostasis results in common neuropsychiatric phenotypes. Nature.

[b63-arh-34-1-106] Richardson DP, Byrnes ML, Brien JF (2002). Impaired acquisition in the water maze and hippocampal long-term potentiation after chronic prenatal ethanol exposure in the guinea-pig. European Journal of Neuroscience.

[b64-arh-34-1-106] Samudio-Ruiz SL, Allan AM, Sheema S, Caldwell KK (2010). Hippocampal N-methyl-D-aspartate receptor subunit expression profiles in a mouse model of prenatal alcohol exposure. Alcoholism: Clinical and Experimental Research.

[b65-arh-34-1-106] Samudio-Ruiz SL, Allan AM, Valenzuela CF (2009). Prenatal ethanol exposure persistently impairs NMDA receptor-dependent activation of extracellular signal-regulated kinase in the mouse dentate gyrus. Journal of Neurochemistry.

[b66-arh-34-1-106] Sanderson JL, Partridge LD, Valenzuela CF (2009). Modulation of GABAergic and glutamatergic transmission by ethanol in the developing neocortex: An in vitro test of the excessive inhibition hypothesis of fetal alcohol spectrum disorder. Neuropharmacology.

[b67-arh-34-1-106] Sathyan P, Golden HB, Miranda RC (2007). Competing interactions between micro-RNAs determine neural progenitor survival and proliferation after ethanol exposure: Evidence from an ex vivo model of the fetal cerebral cortical neuroepithelium. Journal of Neuroscience.

[b68-arh-34-1-106] Schneider ML, Moore CF, Barnhart TE (2005). Moderate-level prenatal alcohol exposure alters striatal dopamine system function in rhesus monkeys. Alcoholism: Clinical and Experimental Research.

[b69-arh-34-1-106] Servais L, Hourez R, Bearzatto B (2007). Purkinje cell dysfunction and alteration of long-term synaptic plasticity in fetal alcohol syndrome. Proceedings of the National Academy of Sciences of the United States of America.

[b70-arh-34-1-106] Shen RY, Choong KC (2006). Different adaptations in ventral tegmental area dopamine neurons in control and ethanol exposed rats after methylphenidate treatment. Biological Psychiatry.

[b71-arh-34-1-106] Thomas JD, Garcia GG, Dominguez HD, Riley EP (2004). Administration of eliprodil during ethanol withdrawal in the neonatal rat attenuates ethanol-induced learning deficits. Psychopharmacology (Berl).

[b72-arh-34-1-106] Thompson BL, Levitt P, Stanwood GD (2009). Prenatal exposure to drugs: Effects on brain development and implications for policy and education. Nature Reviews Neuroscience.

[b73-arh-34-1-106] Toso L, Roberson R, Woodard J (2006). Prenatal alcohol exposure alters GABA(A)alpha5 expression: A mechanism of alcohol-induced learning dysfunction. American Journal of Obstetrics and Gynecology.

[b74-arh-34-1-106] Vaglenova J, Pandiella N, Wijayawardhane N (2008). Aniracetam reversed learning and memory deficits following prenatal ethanol exposure by modulating functions of synaptic AMPA receptors. Neuropsychopharmacology.

[b75-arh-34-1-106] Valenzuela CF, Partridge LD, Mameli M, Meyer DA (2008). Modulation of glutamatergic transmission by sulfated steroids: Role in fetal alcohol spectrum disorder. Brain Research Reviews.

[b76-arh-34-1-106] Valles S, Felipo V, Montoliu C, Guerri C (1995). Alcohol exposure during brain development reduces 3H-MK-801 binding and enhances metabotropic-glutamate receptor-stimulated phosphoinositide hydrolysis in rat hippocampus. Life Sciences.

[b77-arh-34-1-106] Wang J, Haj-Dahmane S, Shen RY (2006). Effects of prenatal ethanol exposure on the excitability of ventral tegmental area dopamine neurons in vitro. Journal of Pharmacology and Experimental Therapeutics.

[b78-arh-34-1-106] Webster WS, Walsh DA, McEwen SE, Lipson AH (1983). Some teratogenic properties of ethanol and acetaldehyde in C57BL/6J mice: Implications for the study of the fetal alcohol syndrome. Teratology.

[b79-arh-34-1-106] Weinberg J, Sliwowska JH, Lan N, Hellemans KG (2008). Prenatal alcohol exposure: Foetal programming, the hypothalamic-pituitary-adrenal axis and sex differences in outcome. Journal of Neuroendocrinology.

[b80-arh-34-1-106] Wijayawardhane N, Shonesy BC, Vaglenova J (2007). Postnatal aniracetam treatment improves prenatal ethanol induced attenuation of AMPA receptor-mediated synaptic transmission. Neurobiology of Disease.

[b81-arh-34-1-106] Wijayawardhane N, Shonesy BC, Vaithianathan T (2008). Ameliorating effects of preadolescent aniracetam treatment on prenatal ethanol-induced impairment in AMPA receptor activity. Neurobiology of Disease.

[b82-arh-34-1-106] Yates WR, Cadoret RJ, Troughton EP (1998). Effect of fetal alcohol exposure on adult symptoms of nicotine, alcohol, and drug dependence. Alcoholism: Clinical and Experimental Research.

[b83-arh-34-1-106] Youngentob SL, Glendinning JI (2009). Fetal ethanol exposure increases ethanol intake by making it smell and taste better. Proceedings of the National Academy of Sciences of the United States of America.

[b84-arh-34-1-106] Zhou FC, Fang Y, Goodlett C (2008). Peptidergic agonists of activity-dependent neurotrophic factor protect against prenatal alcohol-induced neural tube defects and serotonin neuron loss. Alcoholism: Clinical and Experimental Research.

[b85-arh-34-1-106] Zhou FC, Sari Y, Powrozek TA (2005). Fetal alcohol exposure reduces serotonin innervation and compromises development of the forebrain along the serotonergic pathway. Alcoholism: Clinical and Experimental Research.

[b86-arh-34-1-106] Zucca S, Valenzuela CF (2010). Low concentrations of alcohol inhibit BDNF-dependent GABAergic plasticity via L-type Ca2+ channel inhibition in developing CA3 hippocampal pyramidal neurons. Journal of Neuroscience.

